# Circulating exosomes repair endothelial cell damage by delivering miR‐193a‐5p

**DOI:** 10.1111/jcmm.16202

**Published:** 2020-12-22

**Authors:** Chang Cao, Bo Wang, Junnan Tang, Jimin Zhao, Jiacheng Guo, Qianqian Guo, Xiaoting Yue, Zenglei Zhang, Gangqiong Liu, Hui Zhang, Yunzhe Wang, Jinying Zhang

**Affiliations:** ^1^ Department of Cardiology First Affiliated Hospital of Zhengzhou University Zhengzhou Henan China; ^2^ Key Laboratory of Cardiac Injury and Repair of Henan Province Zhengzhou Henan China; ^3^ Department of Pathophysiology School of Basic Medical Sciences Zhengzhou University Zhengzhou Henan China

**Keywords:** exosomes, microRNAs, oxidative damage

## Abstract

Circulating exosomes delivering microRNAs are involved in the occurrence and development of cardiovascular diseases. How are the circulating exosomes involved in the repair of endothelial injury in acute myocardial infarction (AMI) convalescence (3‐7 days) was still not clear. In this study, circulating exosomes from AMI patients (AMI‐Exo) and healthy controls (Normal‐Exo) were extracted. *In vitro* and *in vivo*, our study showed that circulating exosomes protected endothelial cells (HUVECs) from oxidative stress damage; meanwhile, Normal‐Exo showed better protective effects. Through the application of related inhibitors, we found that circulating exosomes shuttled between HUVECs via dynamin. Microarry analysis and qRT‐PCR of circulating exosomes showed higher expression of miR‐193a‐5p in Normal‐Exo. Our study showed that miR‐193a‐5p was the key factor on protecting endothelial cells *in vitro* and *in vivo*. Bioinformatics analyses found that activin A receptor type I (ACVR1) was the potential downstream target of miR‐193a‐5p, which was confirmed by ACVR1 expression and dual‐luciferase report. Inhibitor of ACVR1 showed similar protective effects as miR‐193a‐5p. While overexpression of ACVR1 could attenuate protective effects of miR‐193a‐5p. To sum up, these findings suggest that circulating exosomes could shuttle between cells through dynamin and deliver miR‐193a‐5p to protect endothelial cells from oxidative stress damage via ACVR1.

## 1 INTRODUCTION

Cardiovascular disease has high morbidity and mortality rates worldwide.^1^ Acute myocardial infarction (AMI) is one of the most common and serious vascular diseases, which can gradually develop into chronic heart failure (CHF), and its prevalence is increasing year by year. Clinical guidelines recommend the prompt treatment for AMI patients, but there are still many AMI patients cannot receive treatment until the disease convalescence (3‐7 days after AMI). The researches on endothelial cell injury and its repair mechanism have always been an academic hotspot. However, there is still a lack of effective treatment methods to reverse endothelial function damage and promote angiogenesis. Therefore, it is of great significance to explore the mechanism of occurrence, development and repair of endothelial cell injury, and to find effective new therapeutic targets.

Exosomes are a group of vesicle‐like bodies between 30 and 150 nm in diameter, which are actively secreted by cells. They have a lipid bilayer subcellular structure on the outside and contain proteins similar to their cellular origin, as well as bioactive substances such as lipids, coding or non‐coding RNAs.^2^ Exosomes play an important role in intercellular communication and other processes. Under different pathophysiological conditions, exosomes secreted by cells may undergo diverse changes.^3^ Exosomes can directly stimulate recipient cells by interacting with receptors. Also, it can regulate the function of recipient cells through transporting plasma membrane receptors, functional proteins, mRNA, microRNAs and organelles.^4^, ^5^ During the transport process, exosomes can protect the bioactive substances from degradation and dilution in the extracellular environment.^6^

Circulating exosomes are involved in the occurrence and development of cardiovascular diseases, suggesting that they can be used as an important basis for the diagnosis and possible treatment of cardiovascular disease.^7^ Previous studies reported that heart failure patients showed an increasing level of miR‐192, miR‐194, miR‐34a and other microRNAs related to P53 pathway in circulating exosomes, indicating that microRNAs could be used as predictors of heart failure after myocardial infarction.^8^ Jakob et al. reported that miR‐126 in exosomes derived from endothelial cells could participate in angiogenesis via regulating VEFG signal response and played a role in evaluating the prognosis of patients with cardiovascular disease and the effect of clinical interventions.^9^ In AMI patients, HIF‐1α was involved in the regulation of hypoxia‐induced autophagy of myocardial cells, and it could increase the expression of miR‐30a in serum exosomes.^10^ Ge et al. reported that exosomes from patients with myocardial ischaemia could promote angiogenesis through miR‐939‐5p by targeting iNOS‐NO pathway.^11^ However, it is still not clear that how circulating exosomes involve in the repair of endothelial cell injury in AMI convalescence.

Therefore, the present study investigated how circulating exosomes protect endothelial cells from H_2_O_2_‐induced oxidative stress damage *in vitro* and balloon injury *in vivo*. We verified the interaction pattern between exosomes and cells *in vitro*. miR‐193a‐5p with its downstream target ACVR1 was found to be a key factor in the protective effects of circulating exosomes.

## 2 MATERIALS AND METHODS

### 2.1 Patients

AMI patients in this study were recruited from the first affiliated hospital of Zhengzhou University (China) and Nanyang central hospital (China). The convalescence period of AMI was defined as 3‐7 days after the occurrence of AMI. AMI was defined according to the 2017 ESC Guidelines.^12^ 5 mL venous whole blood samples were collected for exosomes extraction using ExoQuick kit (System Biosciences, USA). Blood samples were centrifuged at 3000 g for 20 minutes at 4°C. ExoQuick reagent was added to the supernatant in a ratio of 1:4 for 30 minutes. After centrifugation at 1500 g for 30 minutes, sediment was suspended in PBS of equal serum volume, and the purified circulating exosomes solution was stored at −80°C.

### 2.2 Identification of circulating exosomes

The morphological characters of circulating exosomes were observed by transmission electron microscopy (TEM) with uranyl acetate staining. The diameter distribution and concentration of exosomes were analysed using nanoparticle tracking analyzer (NanoSight NS300). BCA kits (Solarbio, China) were used to determine the protein concentration of circulating exosomes. Expression of exosome surface markers were tested by Western blot.

### 2.3 Cell culture

Human umbilical vein endothelial cells (HUVECs) were purchased from American Tissue Culture Collection (ATCC, USA). HUVECs were cultured in cell incubators at 5% CO_2_ and 37°C using endothelial cell medium (ECM, Lonza, Switzerland) kit. Human embryonic kidney 293T cells (293T cells) were purchased from the Type Culture Collection of the Chinese Academy of Sciences (Shanghai, China). 293T were cultured in cell incubators at 5% CO_2_ and 37°C using Dulbecco's Modified Eagle Medium (DMEM, high glucose, Biological Industries, Israel) with 10% foetal bovine serum (Biological Industries, Israel).

### 2.4 CCK‐8 assay

Human umbilical vein endothelial cells (8 × 10^3^ cells per well) were seeded in 96‐well plates. After cell adhesion, medium was replaced by complete ECM medium containing H_2_O_2_ (600 μmol/L) with PBS, Normal‐Exo (100 μg/mL) or AMI‐Exo (100 μg/mL) for 24 hours. CCK‐8 (Dojindo, Japan) reagent 10 μL was added into each well for another 1 hour. Absorbance was measured at 450 nm using a 96‐well microplate reader, and the optical density values represented the survival of HUVECs.

### 2.5 Tube formation assay

Matrigel (Corning, USA) was plated in 96‐well plates and incubated at 5% CO_2_ and 37°C for 2 hours. Then, HUVECs (5 × 10^3^ cells per well) suspended in serum‐free ECM medium containing H_2_O_2_ with PBS, Normal‐Exo (100 μg/mL) or AMI‐Exo (100 μg/mL) were seeded on the Matrigel‐coated 96‐well plates and incubated for 6 hours. Tube formation was evaluated by inverted fluorescence microscope. The total branching length of HUVECs was measured with ImageJ (version 1.52, NIH).

### 2.6 Scratch wound assay

For the scratch wound assay, HUVECs (3 × 10^5^ cells per well) were seeded in six‐well plates After cell adhesion, HUVECs were scraped with 200 μL pipette tip. Then, medium was replaced by serum‐free ECM medium containing H_2_O_2_ (600 μmol/L) with PBS, Normal‐Exo (100 μg/mL) or AMI‐Exo (100 μg/mL). Cell migration was evaluated by inverted fluorescence microscope after 12 hours. The images were processed by ImageJ to mark the scratch areas.

### 2.7 Transwell assay

Human umbilical vein endothelial cells were suspended in serum‐free ECM medium containing H_2_O_2_ (600 μmol/L) with PBS, Normal‐Exo (100 μg/mL) or AMI‐Exo (100 μg/mL). Then mixtures were seeded into the upper chamber of transwell 24‐well plates (Corning, USA) with 8‐μm pore filters for 24 hours. And the lower chamber was filled with complete ECM medium. Cells attached on the lower surface were fixed by 95% methanol for 20 minutes and stained with 0.05% crystal violet for 20 minutes at room temperature. Cell migration was evaluated by inverted fluorescence microscope.

### 2.8 Fluorescent labelling of exosomes

Circulating exosomes were labelled with PKH26 kit (PKH26 Red Fluorescent Cell Linker Kit, Sigma, USA). 100 μL Solution C was added to 50 μL circulating exosomes suspended in PBS. 0.5 μL PKH26 was dissolved in another 100 μL solution C. The above solutions were mixed and incubated for 5 minutes, and 200 μL 5% BSA was added to stop staining. Circulating exosomes labelled with PKH26 were isolated using ExoQuick‐TC kit.

### 2.9 Uptake capacity of circulating exosomes in vitro

Circulating exosomes (100 μg/mL) labelled with PKH26 were added to ECM. After 24 hours, HUVECs incubated with the above ECM were washed with PBS for three times. Fluorescence signal of PKH26 was observed by inverted fluorescence microscope.

### 2.10 Microarray

Circulating exosomes from AMI convalescence patients (n = 6) and healthy control (n = 3) were analysed by Illumina HiSeq 2500 High‐throughput sequencing platform of Genesky Biotechnologies Inc. (Shanghai, China) according to standard methods.

### 2.11 Quantitative real‐time PCR (qRT‐PCR)

Total RNA in exosomes was extracted by TRIzol (Solarbio, China). The miR‐193a‐5p primers were TGGGTCTTTGCGGGCG (Sangon Biotech, China). MiScript SYBR Green PCR Kit (QIAGEN, German) was used to test the relative microRNA levels with QuantStudio 5 real‐time PCR system (Thermo Fisher Scientific, USA).

### 2.12 Transfection of miRNA mimics and plasmid into HUVECs

HUVECs were digested by trypsin and then cultured into a six‐well culture plate with a density of 50‐70%. After cell adhesion, complete ECM was replaced with serum‐free ECM medium. For RNA, miR‐193a‐5p mimics (Sense: 5' UGGGUCUUUGCGGGCGAGAUGA 3', Antisense: 5'AUCUCGCCCGCAAAGACCCAUU 3') and mimic negative control (mimic‐NC, Sense:5' UUCUCCGAACGUGUCACGUTT 3', Antisense: 5' ACGUGACACGUUCGGAGAATT 3') (GenePharma, China) were diluted in 125μL serum‐free ECM medium. For plasmid, miR‐193a‐5p mimics and PEX‐3. ACVR1 plasmid (GenePharma, China) with P3000 regent (Thermo Fisher Scientific, USA) or miR‐193a‐5p mimics and PEX‐3. NC plasmid (GenePharma, China) with P3000 regent were diluted in 125 μL serum‐free ECM medium. Meanwhile, lipofectamine 3000 (Thermo Fisher Scientific, USA) transfection reagent was diluted with 125 μL serum‐free ECM medium. The above solutions were mixed and incubated for 15 minutes at room temperature. The mixtures were added to the serum‐free ECM medium with the working concentration of RNA 100 nmol/L, and plasmid 2500 ng. HUVECs were cultured in cell incubators at 5% CO_2_ and 37°C for 6 hours. The medium in six‐well culture plate was replaced by complete ECM medium. Transfected HUVECs were further cultured for 24 hours.

### 2.13 Transfection of miRNA mimics into circulating exosomes

Circulating exosomes were diluted to 0.5 μg/μL using gene pulser electroporation buffer (Bio‐Rad, USA), and 400 μL exosomes were added to the electroporation cup (0.4 mm, Bio‐Rad, USA). 10 μL dilution of microRNAs mimics or its negative control (NC) was added to the circulating exosomes in electroporation cup. The Gene Pulser Xcell electroporation system (Bio‐Rad, USA) was used with the parameters of 400 mF, 125 μf and 10‐15 ms for three times. The mixtures were incubated on ice for 5 minutes after each electroporation.^13^ After the electroporation was completed, the mixtures were added to 2 mL 1% BSA PBS solution. Circulating exosomes transfected with mimics or mimic‐NC were isolated using ExoQuick‐TC kit.

### 2.14 Animals selection and experimental ethics

Male Sprague Dawley (SD) rats (6‐8 weeks old) purchased from Beijing Vital River Laboratory Animal Technology Co. Ltd. (Beijing, China) were raised to weight 320‐350 g for experiment. The rats were raised in the standard household animal experiment centre in Zhengzhou University (Zhengzhou, China). The drinking water and standard feed for rats were strictly sterilized, and at least 7 days prior to the start of the experiment were used to recover the rats from transport stress. The surgery protocol was designed to minimize animal pain. Experiments were approved by ethics committee of the first affiliated hospital of Zhengzhou University.

### 2.15 Establishment of carotid artery balloon injury model in rats

10% chloral hydrate (100 g/mL) was used to anaesthetize rats by intraperitoneal injection. The skin was cut off along the median line, and the salivary glands were dissociated from the median line after the subcutaneous tissue was separated. The common carotid artery and its branches internal and external carotid artery passed between torn masseter and sternocleidomastoid. The arteries were completely separated for the next step. The guide wire was rapidly inserted into the incision on external carotid artery to guide 1.5 mm × 20 mm balloon catheter (MicroPort, China) into the common carotid artery. Balloon was inflated to pressure 6atm. After the balloon was pulled, it was evacuated in vacuum. The above steps were repeated three times. Circulating exosomes (400 μg per rat) were dropped on the surface of the arteries. Muscle and skin were sutured with surgical sutures of 5‐0 and 4‐0, respectively. Rats were placed on heating pad until fully awake and then returned to cages.

### 2.16 Histological analysis

Following anaesthesia, the artery was excised and immediately placed in 4% paraformaldehyde at room temperature for 24 hours. The arterial specimens were embedded in paraffin and cut into 4 μm sections. Serial artery sections were stained with haematoxylin and eosin (HE, Solarbio, China). Images were analysed using ImageJ.

### 2.17 Western blot

Cells and exosomes were lysed with ice‐cold RIPA lysis buffer (Solarbio, China). Samples (10 μg) were loaded on to sodium dodecyl sulphate/polyacrylamide gel electrophoresis (SDS/PAGE) and transferred to polyvinylidene fluoride membranes (Millipore, USA). The membranes were blocked with 5% milk in Tris‐buffered saline containing Tween‐20 (TBST) for 2 hours at room temperature. Then, the membranes were incubated with primary antibodies against β‐actin (Zhongshan Goldenbridge Biotechnology, China, 1:2000), GADPH (Hangzhou Goodhere Biotechnology, China, 1:2000), HSP70 (Abcam, USA, 1:1000), Alix (Cell Signaling Technology, USA, 1:1000) and ACVR1 (ProteinTech, China, 1:1000) at 4°C overnight. The membranes were incubated with secondary antibody (Cell Signaling Technology, USA) for 2 hours at room temperature. Horseradish peroxidase kit (Thermo Fisher Scientific, USA) was used to conject secondary antibody, and proteins were detected by Amersham Imager 600 (GE Healthcare Life Sciences, USA). Densitometric quantification of bands intensity were analysed by ImageJ.

### 2.18 Dual‐luciferase report assay

The Firefly luciferase and Renilla luciferase reporter gene detection system was used to verify the direct effect of miR‐193a‐5p on target genes. The 3'‐UTR of the human ACVR1 sequence containing the predicted miR‐193a‐5p‐binding sites, or its mutant was loaded into plasmid vectors (pmirGLO‐ACVR1 WT or pmirGLO‐ACVR1 MUT) (GenePharma, China). 293T cells were co‐transfected with above plasmid vectors, respectively, and human miR‐193a‐5p mimics or its NC. After 48 hours, a dual‐luciferase report assay (Promega, USA) following manual was used to measure the firefly luciferase and renilla luciferase activities. Luciferase results shown as relative light units (firefly luciferase activity divided by renilla luciferase activity) were detected with multi‐mode microplate readers (Molecular Devices, USA).

### 2.19 Statistical analysis

All the experiments were repeated at least three times. Average data were shown as mean ± standard errors. Comparisons between two groups were conducted by two‐tailed *t* test. One‐way ANOVA tests were used for comparison among three or more groups with Bonferroni post hoc correction (version 7.0, GraphPad Software). A *P* < .05 was regarded as statistically different.

## 3 RESULTS

### 3.1 Biological identification of circulating exosomes

**FIGURE 1:**
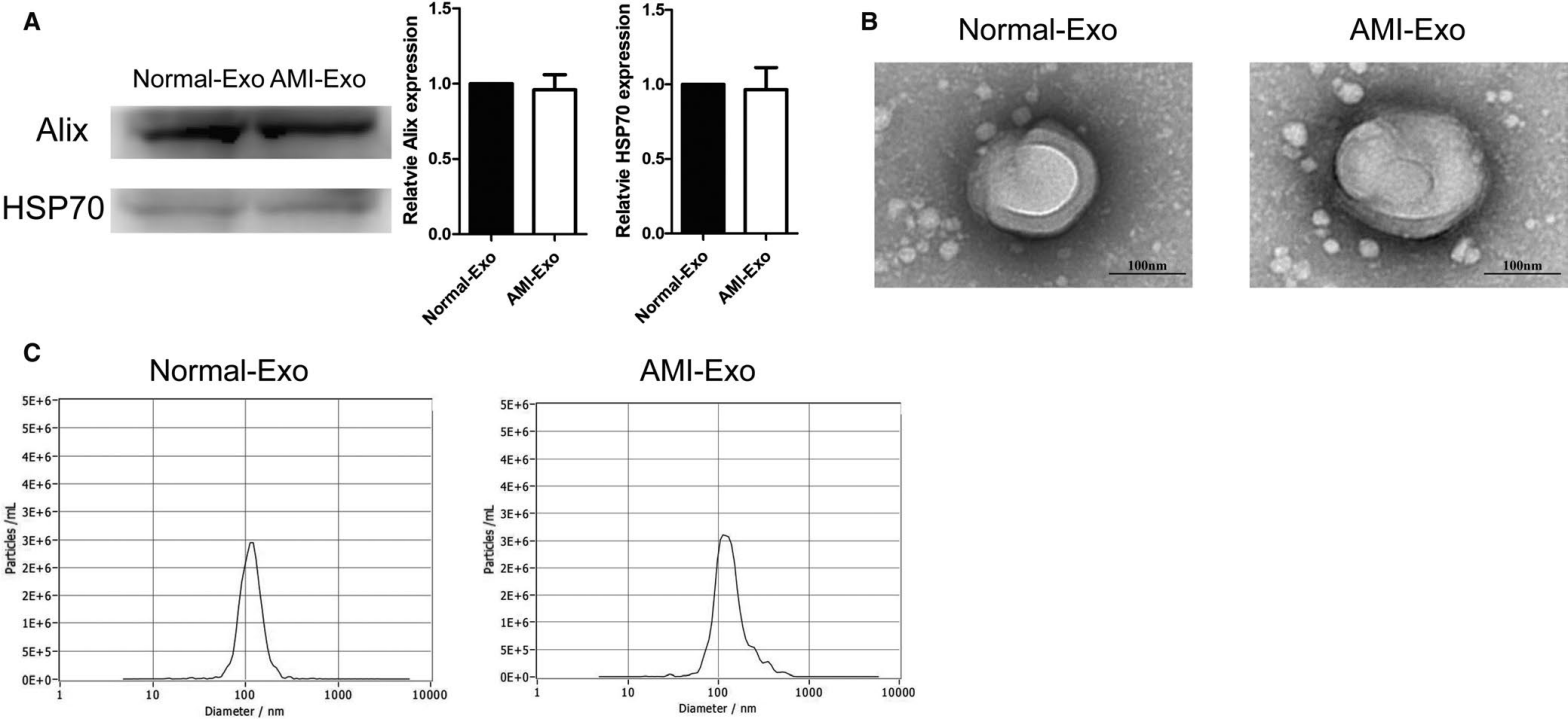
Biological identification of circulating exosomes from patients. (A) HSP70, and Alix expression levels in Normal‐Exo and AMI‐Exo (n = 3). (B) Exosome scanning results of transmission electron microscopy (TEM). Bars indicated 100 nm. (C) The particle diameter distribution ratio tested by Nanoparticle tracking analysis of Normal‐Exo and AMI‐Exo

We harvested exosomes from 12 AMI patients and 9 healthy controls, and the baseline characteristics of these patients were shown in Table 1. Western blot was used to detect the specific protein markers of circulating exosomes, including HSP70, Alix (Figure 1A). There was no statistically significant differences in the expression of markers of circulating exosomes between the two groups. Transmission electron microscopy (TEM) showed that circulating exosomes were in bubble‐like balloon shape with diameter distribution of 50‐150 nm (Figure 1B), which was consistent with the results of Nanoparticle tracking analysis (NTA) (Figure 1C). The two groups of circulating exosomes were similar in physical characters such as morphology and diameter distribution.

**TABLE 1:** The baseline characteristics of AMI convalescence patients and healthy controls

Characteristics	AMI convalescence patients	Healthy controls	*P* value
Age (y)	51.2 ± 9.3	46.3 ± 11.0	.31
Sex (Male: Female)	8: 4	6:3	1.0
Body mass index (BMI)	25.9 ± 2.9	24.4 ± 1.1	.14
Systolic pressure (mm Hg)	134.33 ± 24.02	130.56 ± 15.29	.68
Diastolic pressure (mm Hg)	82.58 ± 15.11	75.56 ± 12.10	.26
Glucose (mmol/L)	7.87 ± 2.92	6.65 ± 1.44	.27
LDL‐C (mmol/L)	2.79 ± 0.87	2.64 ± 0.73	.68
Troponin T positive (%)	11 (91.7)	0 (0)	<.001

### 3.2 Circulating exosomes protected endothelial cells from oxidative stress damage

**FIGURE 2:**
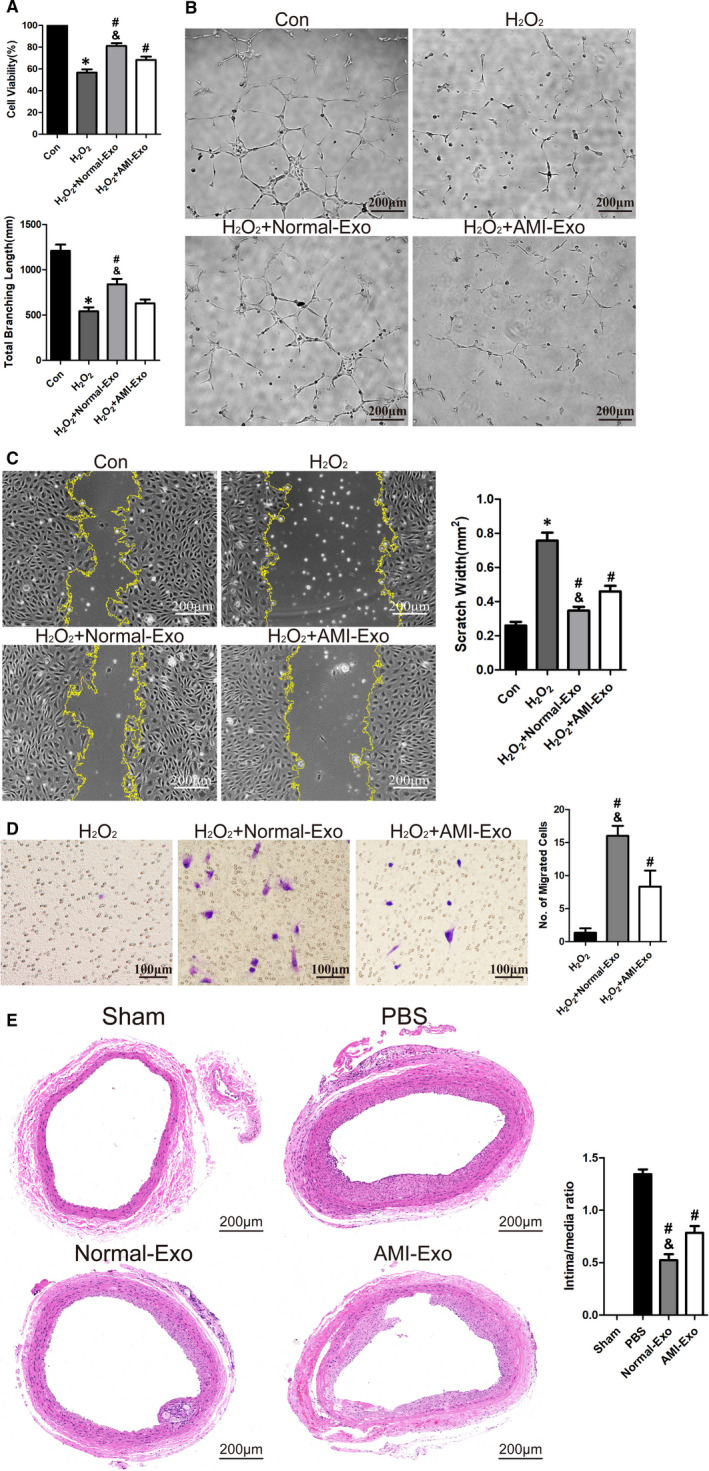
Circulating exosomes attenuated H_2_O_2_‐induced oxidative stress damage. (A) H_2_O_2_ (600 μmol\L) together with circulating exosomes (Normal‐Exo or AMI‐Exo, 100 μg/mL) were used to treat HUVECs for 24 h. CCK‐8 assay to test protective effect of circulating exosomes (n = 3). (B) HUVECs were treated as shown in (A) for 6 h, tube formation assay measured by total branching length to test protective effect of circulating exosomes (n = 3). Bars indicate 200 μm. (C) HUVECs were treated as shown in (A) for 12 h, scratch wound assay measured by scratch width to test protective effect of circulating exosomes (n = 3). Bars indicated 200 μm. (D) HUVECs were treated as shown in (A) for 24 h, transwell assay measured by migrated cells number to test protective effect of circulating exosomes (n = 3). Bars indicated 100 μm. (E) PBS or Circulating exosomes (Normal‐Exo or AMI‐Exo) were dropped on the surface of carotid artery in rat common carotid artery balloon injury model. Representative images of artery cross sections with HE staining and statistical results of intima/media ratio (n = 5). Bars indicated 200 μm. The data are expressed as the mean ± SEM. *indicated *P* < .05, compared with the control group; # indicated *P *< .05, compared with the H_2_O_2_ group in (A‐D); # indicated *P *< .05, compared with the PBS group in (E); & indicated *P *< .05, compared with the H_2_O_2_+AMI‐Exo group in (A‐D); & indicated *P *< .05, compared with the AMI‐Exo group in (E)

Circulating exosomes play an important role in the intercellular materials and information exchange. Previous researches have reported that serum exosomes can promote angiogenesis of endothelial cell, which is a crucial factor in the development of cardiovascular disease.^14^, ^15^ In order to verify whether circulating exosomes can reduce the oxidative stress damage induced by H_2_O_2_, HVUECs were treated with H_2_O_2_ (600 μmol/L) and circulating exosomes derived from the two groups. In CCK8 assay, Normal‐Exo (100 μg/mL) and AMI‐Exo (100 μg/mL) protected HUVECs from H_2_O_2_ after 24 hours. Besides, Normal‐Exo showed better protective effect (Figure 2A). In tube formation assay, total branching length of HUVECs treated with AMI‐Exo (100 μg/mL) was lower than that treated with Normal‐Exo (100 μg/mL), which meant that AMI‐Exo had a weaker protective effect than Normal‐Exo, in terms of vascular formation ability (Figure 2B). In addition, the protective effects of exosomes on cell migration also varied between the two groups.

Both scratch wound assay and transwell assay showed that circulating exosomes (100 μg/mL) could protect HUVECs from H_2_O_2_‐induced oxidative stress damage on cell migration. The scratch wound area of HUVECs treated with Normal‐Exo was smaller, and the migrated number of HUVECs treated with Normal‐Exo was also (Figure 2C‐D). To sum up, circulating exosomes of both group could protect endothelial cells from oxidative stress damage mediated by H_2_O_2_. However, Normal‐Exo had prior protective effects than AMI‐Exo in vivo. And the vascular protective effect of circulating exosomes was further confirmed in the rat model of balloon injury. 14 days after surgery, the quantitative analysis of intima/media ratios showed that both Normal‐Exo (400 μg/rat) and AMI‐Exo (400 μg/rat) could significantly reduce neointimal hyperplasia caused by mechanical damage, while Normal‐Exo showed better protective effects (Figure 2E).

### 3.3 Circulating exosomes shuttled between cells through dynamin

**FIGURE 3:**
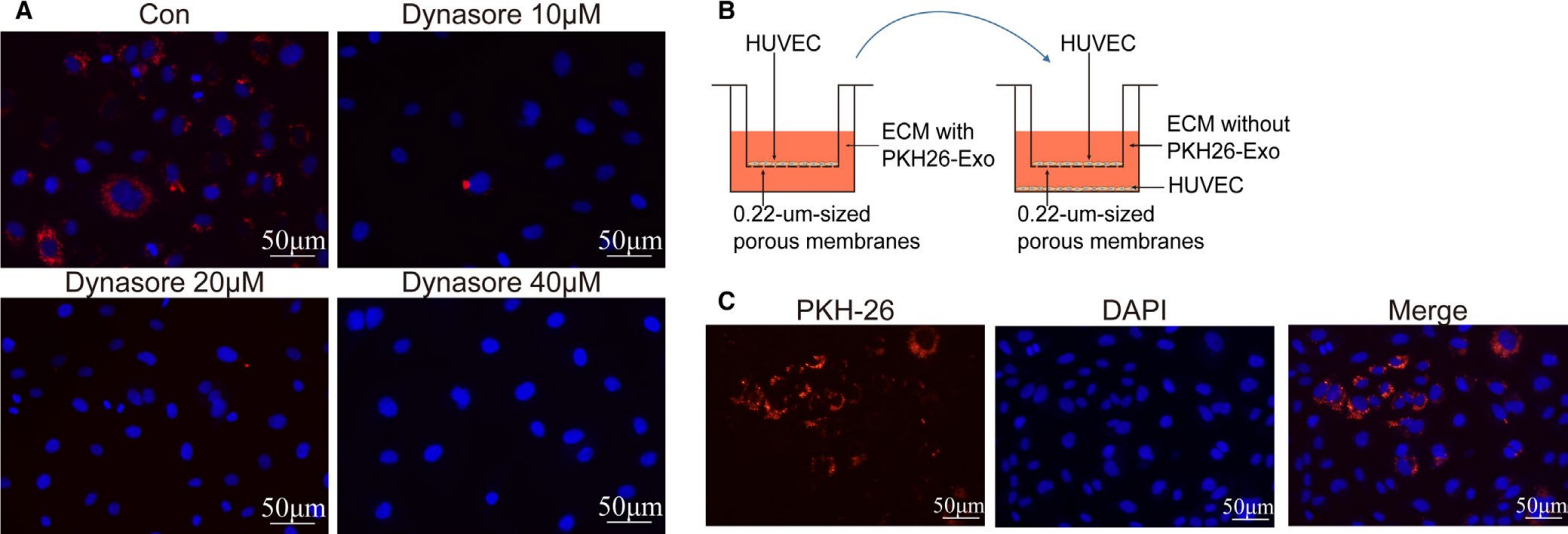
Circulating exosomes shuttled between cells through dynamin. (A) The distribution of stained exosomes in HUVECs treated with inhibitor of dynamin, dynasore (at concentration 10, 20 and 40 μmol/L) for 24 h. Red: exosomes stained with PKH26, blue: DAPI. Bars indicated 50 μm. (B) Schematic diagram of transwell system. HUVECs seeded on the upper chamber, were co‐cultured with exosomes (100 μg/mL) stained by PKH26 for 24 h. Then, moving the upper chamber to another transwell system, in which the lower chamber was seeded with HUVECs. (C) Representative fluorescent images of HUVECs in lower chambers. Red: exosomes stained with PKH26, blue: DAPI. Bars indicated 50 μm

Circulating exosomes interact with cells in a variety of ways, among which the most convincing hypothesis is that exosomes release their contents after being ingested by cells.^16^ In order to verify whether the uptake of circulating exosomes via dynamin, HUVECs were co‐cultured with dynamin inhibitor, dynasore. And circulating exosomes (100 μg/mL) were stained by PKH‐26. After 24 hours, fluorescence microscopy showed that compared with the control group, dyansore had a significant, dose‐dependent inhibitory effect on the uptake of circulating exosomes, suggesting that circulating exosomes were ingested by HUVECs through dynamin (Figure 3A). Transwell system was used to verify this function. And the results showed that HUVECs in lower chambers could ingest stained exosomes without direct contact with HUVECs in upper chambers (Figure 3B‐C), suggesting that circulating exosomes can shuttle among cells.

### 3.4 miR‐193a‐5p might be the key factor in protective effects of circulating exosomes

**FIGURE 4:**
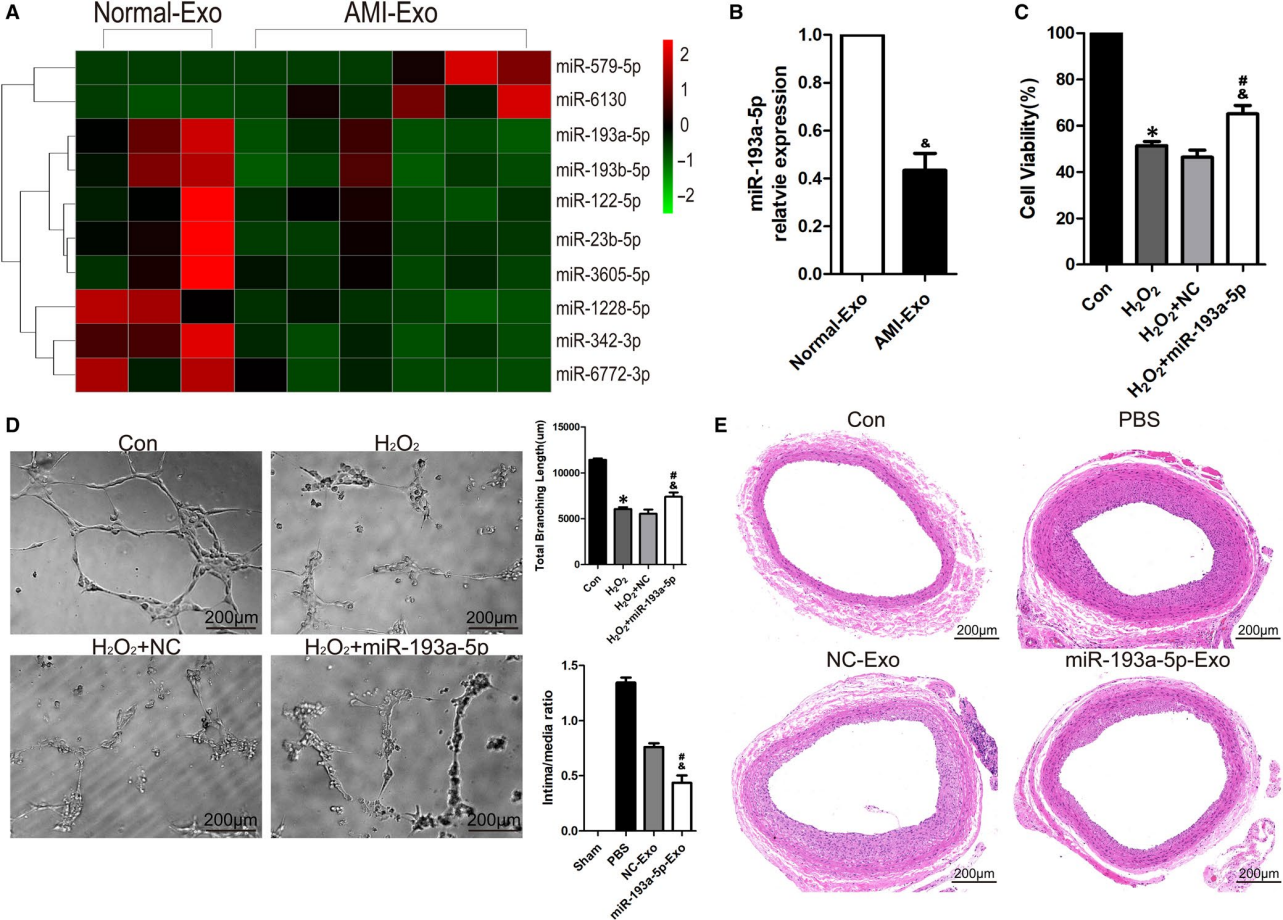
miR‐193a‐5p protected endothelial cells. (A) The heat map of differential expression microRNAs in Normal‐Exo (n = 3) and AMI‐Exo (n = 6). The red dots: up‐regulated microRNAs. Green dots: down‐regulated microRNAs. (B) qRT‐PCR was performed to analyse the miR‐193a‐5p levels in circulating exosomes (Normal‐Exo, AMI‐Exo) (n = 3) (C) HUVECs were transfected with mimics negative control (NC) or mimics of miR‐193a‐5p for 24 h. H_2_O_2_ (600 μmol\L) were used to treat transfected HUVECs for 24 h. CCK‐8 assay to test protective effect of miR‐193a‐5p (n = 3). (D) HUVECs were transfected with mimics negative control (NC) or mimics of miR‐193a‐5p for 24 h. H_2_O_2_ (600 μmol/L) were used to treat transfected HUVECs for 6h, tube formation assay measured by total branching length to test protective effect of miR‐193a‐5p (n = 3). Bars indicate 200 μm. (E) AMI‐Exo transfected with NC or mimics of miR‐193a‐5p were dropped on the surface of carotid artery in rat common carotid artery balloon injury model. Representative images of artery cross sections with HE staining and statistical results of intima/media ratio (n = 5). Bars indicated 200 μm. The data are expressed as the mean ± SEM. * indicated *P* < .05, compared with the control group; # indicated *P* < .05, compared with the H_2_O_2_ group in (A‐C); # indicated *P* < .05, compared with the PBS group in (D); & indicated *P* < .05, compared with the Normal‐Exo group (B); & indicated *P* < .05, compared with the H_2_O_2_+NC group in (C‐D); & indicated *P* < .05, compared with the NC‐Exo group in (E)

All experiments mentioned above revealed that compared with AMI‐Exo, Normal‐Exo had a more obvious vascular protection effect. And the contents of circulating exosomes, such as proteins, microRNAs and lnc‐RNA, might be responsible for their protective effects. To further investigate the underlying mechanism, we explored the microRNAs of the two groups exosomes. After a microarray analysis, the differently expressed microRNAs in the two groups were shown in the heat map (Figure 4A). qRT‐PCR confirmed differential expression of miR‐193a‐5p in two groups (Figure 4B). Mimics of miR‐193a‐5p were transfected into HUVECs. Then, transfected HUVECs were treated with H_2_O_2_ (600 μmol/L) for 24 hours, and CCK‐8 assay confirmed that miR‐193a‐5p showed obvious protective effect on HUVECs when compared with negative control (Figure 4B). miR‐193a‐5p is an important target in various diseases, and it plays different roles in different models.^17^ To confirm whether miR‐193a‐5p could protect vascular formation ability of HUVECs, miR‐193a‐5p mimics and mimics negative control were transfected into HUVECs for function analyses. Tube formation assay showed the same results as previous CCK‐8 assay (Figure 4C), suggesting that miR‐193a‐5p directly protected HUVECs from H_2_O_2_‐induced oxidative stress damage. In rat carotid artery balloon injury models, miR‐193a‐5p mimics and negative control were transfected into AMI‐Exo through electroporation to confirm that miR‐193a‐5p did play a role through circulating exosomes in vivo. The quantitative analysis of intima/media ratios showed that miR‐193a‐5p was essential to the protective effects of exosomes (400 μg/rat). (Figure 4D). To sum up, miR‐193a‐5p, delivered by circulating exosomes, played an important role in promoting the repair of endothelial damage in vitro and in vivo.

### 3.5 AVCR1 as a target gene of miR‐193a‐5p were involved in protecting endothelial cells

**FIGURE 5:**
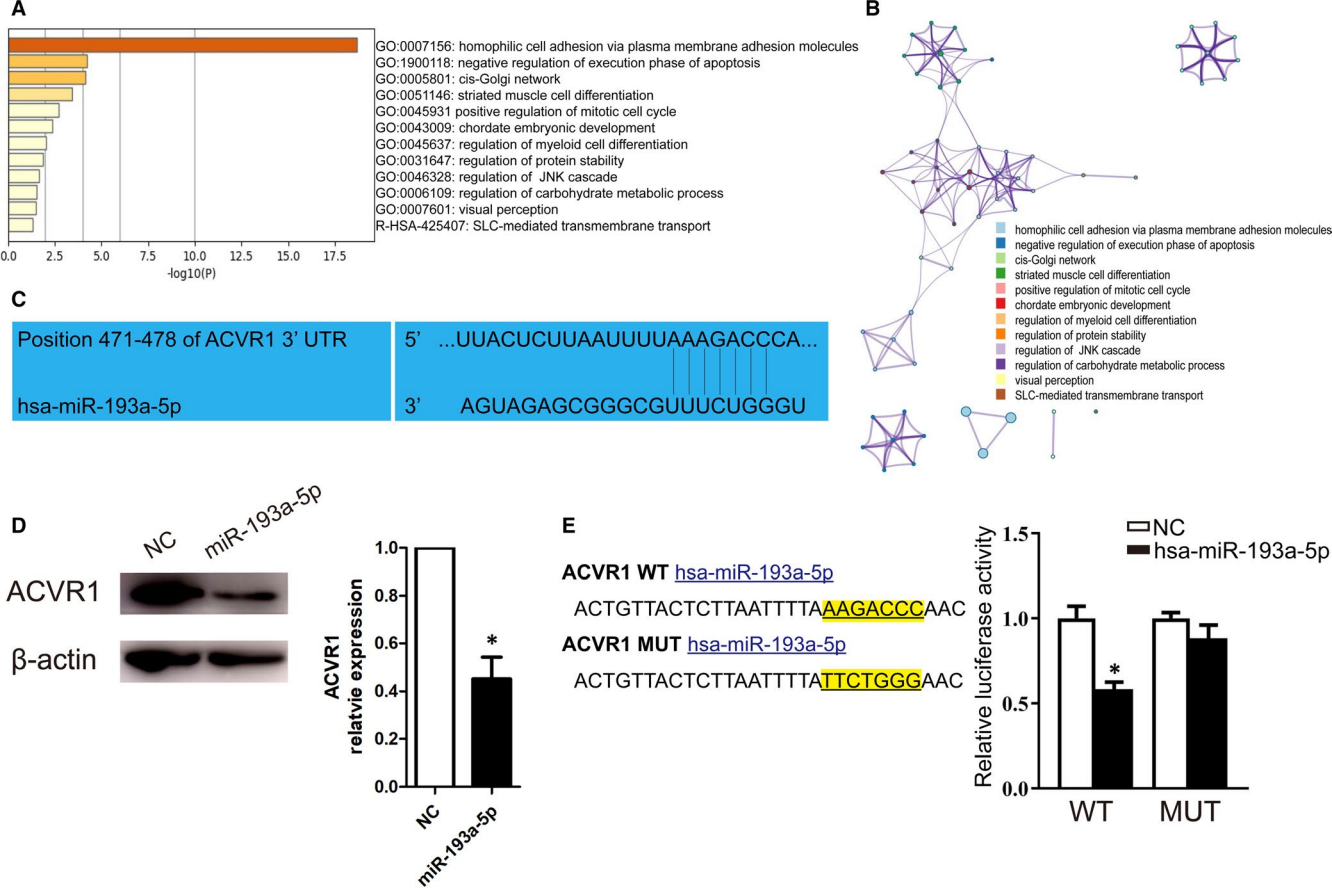
Downstream target of miR‐193a‐5p. (A) Go analysis and (B) KEGG analysis of potential downstream targets of miR‐193a‐5p. (C) The predicted consequential pairing of ACVR1 (top) and miR‐193a‐5p (bottom). (D) The ACVR1 and β‐actin expression levels in HUVECs transfected with NC or miR‐193a‐5p (n = 3). (E) Plasmid vectors of human ACVR1 3'‐UTR or its mutation were transfected into 293T cells followed by miR‐193a‐5p mimics or its NC. Statistical results of dual‐luciferase activity assay (n = 3). The data are expressed as the mean ± SEM. * indicated *P* < .05, compared with the NC group

Bioinformatics analyses including GO and KEGG were applied to screen potential downstream targets of miR‐193a‐5p. The results showed that plasma membrane adhesion molecules managing homophilic cell adhesion were the most correlated targets (Figure 5A‐B). Among these predicted target genes, activin A receptor type I (ACVR1), also known as activin receptor‐like kinase 2 (ALK2), had been intensively studied in cell function and proliferation.^18^ To verify whether the expression of ACVR1 was affected by miR‐193a‐5p, Western blot was used to detect the expression of ACVR1 in HUVECs transfected with mimics of miR‐193a‐5p. The result showed that mimics of miR‐193a‐5p significantly inhibited the expression of ACVR1 (Figure 5D). Dual‐luciferase report confirmed that ACVR1 was the target gene of miR‐193a‐5p (Figure 5E).

**FIGURE 6:**
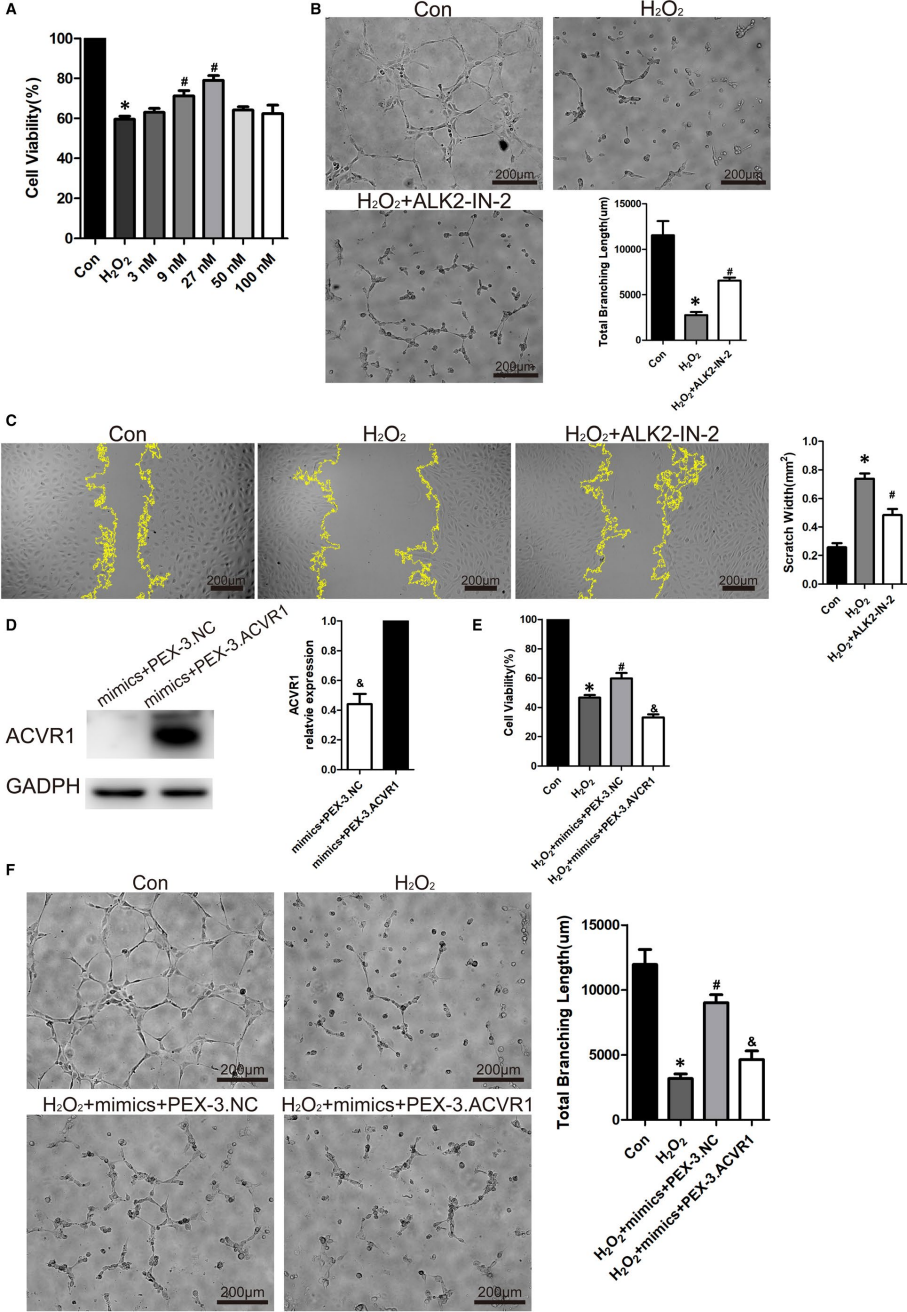
ACVR1 as a key factor for the protective effects of miR‐193a‐5p. (A) H_2_O_2_ (600 μmo/L) together with the inhibitor of ACVR1 (ALK2‐IN‐2) (concentration at 3, 9, 27, 50 and 100 nmol/L) were used to treat HUVECs for 24 h. CCK‐8 assay to test vascular protective effect of ACVR1 (ALK2‐IN‐2) (n = 3). (B) HUVECs treated with H_2_O_2_ (600 μmol/L) together with ALK2‐IN‐2 (27 nmol/L) were treated for 6 h, tube formation assay measured by total branching length to test protective effect of ALK2‐IN‐2 (n = 3). Bars indicate 200 μm. (C) HUVECs treated with H_2_O_2_ (600 μmol/L) together with ALK2‐IN‐2 (27 nmol/L) were treated for 12 h, scratch wound assay measured by scratch width to test protective effect of circulating exosomes (n = 3). Bars indicated 200 μm. (D) HUVECs were transfected with mimics of miR‐193a‐5p + negative control plasmid (mimics+PEX‐3.NC) or mimics of miR‐193a‐5p + ACVR1 overexpression plasmid (mimics+PEX‐3. ACVR1) for 24 h. Western blot to test the expression of ACVR1 and GADPH in HUVECs after transfection. (E) HUVECs were treated as shown in (D) were treated with H_2_O_2_ (600 μmol/L) for 24 h. CCK‐8 assay to test protective effect (n = 3). (F) HUVECs were treated as shown in (D) were treated with H_2_O_2_ (600 μmol/L) for 6 h, tube formation assay measured by total branching length to test protective effect (n = 3). Bars indicate 200 μm. The data are expressed as the mean ± SEM. *indicated *P* < .05, compared with the control group. #indicated *P* < .05, compared with the H_2_O_2_ group; & indicated *P* < .05, compared with the mimics+PEX‐3.ACVR1 group in (D). & indicated *P* < .05, compared with the H_2_O_2_ + mimics + PEX‐3.NC group in (E‐F)

As miR‐193a‐5p could inhibit the expression of ACVR1, we further explored whether the inhibition of ACVR1 was responsible for the protective effect of miR‐193a‐5p. ALK2‐IN‐2 (MCE) is a potent and selective inhibitor of ACVR1 with an IC50 of 9nM, and over 700‐fold selectivity against ALK3. In CCK8 assay, inhibitor of ACVR1 showed the same protective effects as miR‐193a‐5p in HUVECs, and the effect is concentration dependent (Figure 6A). Tube formation assay and scratch wound assay also confirmed that inhibiting ACVR1 (ALK2‐IN‐2 at 27 nmol/L) could reduce the injury induced by oxidative stress to endothelial cell functions (Figure 6B‐C), suggesting the important role of ACVR1 in endothelial cell pathophysiological changes. Transfection of microRNAs mimics and its target gene overexpressed plasmid into cells is an important experimental process to verify the functional relationship between microRNAs and its target genes. After the transfection of mimics and plasmid, Western blot showed that ACVR1 was highly expressed when HUVECs were transfected with PEX‐3. ACVR1 compared with PEX‐3. NC (Figure 6D). Meanwhile, HUVECs were used to conduct relevant experiments 24h after the synchronous transfection. Both CCK8 assay and tube formation assay showed that overexpression of ACVR1, a target gene of miR‐193a‐5p, could reverse the vascular protective effects of miR‐193a‐5p (Figure 6E‐F), indicating that miR‐193a‐5p protected HUVECs from H_2_O_2_‐induced oxidative stress damage via targeting ACVR1.

## 4 DISCUSSION

The components of circulating exosomes varied in different stages of disease, suggesting that they could influence the occurrence and development of disease.^19^, ^20^, ^21^ Ge et al. enrolled patients who were suspected of myocardial ischaemia in past three months and found that exosomes from myocardial ischaemia patients could promote angiogenesis.^11^ In our present study, we strictly selected the time after the onset of myocardial infarction. And we found that exosomes from AMI convalescence patients and healthy control could promote the repair of HUVECs damaged by H_2_O_2_. And circulating exosomes from healthy control had better protective effects than that from AMI patients in vitro and in vivo (Figure 2).

There are several mechanisms related to the interaction between cells with exosomes, including (a) exosomes recognize the surface receptors of the target cells by ligand‐receptor binding, leading to signal transduction; (b) exosomes directly fuse with target cells to achieve intercellular receptor transfer and signal transduction; (c) exosomes fuse with target cells, releasing the content proteins into target cells to exert biological functions; and (d) exosomes transmit genetic information to target cells through mRNA, microRNAs or other transcription factors.^22^, ^23^, ^24^ Among these related mechanisms, the interaction of exosomes and target cells is a crucial step in producing biological effects. Hallbeck et al. reported that the uptake of exosomes in Alzheimer's disease is dynamin‐dependent, which can be blocked by its inhibitor dynasore.^25^ Furthermore, exosomes propagate the toxic materials between cells, spreading pathology in the Alzheimer brain.^26^, ^27^ Our study showed that circulating exosomes could be uptaken by cells and undertake the cell‐cell communications, while maintaining membrane structure and internal substances, indicating the possible mechanism of the protective effects of circulating exosomes on oxidative stress damage (Figure 3).

In recent years, microRNAs in exosomes had gradually become a research hotspot. Specific microRNAs have been proven to play an important role in disease development and recovery, which meant that microRNAs could be potential targets for the diagnosis, prevention and treatment of disease.^28^, ^29^ To find out the functional microRNAs in circulating exosomes, microarray analysis and qRT‐PCR were used to investigate the different microRNAs profiles in the AMI‐Exo and Normal‐Exo. Our results showed that the expression of miR‐193a‐5p were significantly down‐regulated in AMI‐Exo, suggesting it might be responsible for the different functions of Norma‐Exo and AMI‐Exo (Figure 4A‐B). With reference to CCK‐8 assay and tube formation assay, miR‐193a‐5p were screened as a key molecule for further study (Figure 4C‐D). MiR‐193a‐5p with its family miR‐193 had been identified to be closely related to the occurrence and development of cardiovascular diseases in several researches.^30^ Chang et al. reported that miR‐193‐3p was up‐regulated in vascular tissues of rat ischaemia‐reperfusion injury.^31^ Also, up‐regulation of miR‐193‐5p was observed in the circulation system of patients with hypertrophic cardiomyopathy.^32^ Meanwhile, miR‐193a‐5p is an important target for a variety of tumour diseases. The overexpression or low expression of miR‐193a‐5p contributes to tumour cell proliferation, invasion, migration, apoptosis and metastasis.^33^ In studies of anti‐tumour drugs, miR‐193a‐5p played a role in protecting cancer cells. Zhang *et al*. reported the silencing of miR‐193a‐5p enhanced the chemosensitivity of prostate cancer cells to docetaxel‐induced apoptosis via HO‐1 in vitro. And depletion of miR‐193a‐5p reduced PC xenograft growth in vivo.^34^ Zhou et al. found that overexpression of miR‐193a‐5p increased the capacity of migration and cisplatin resistance in urothelial cells.^35^

In study of cell derived exosomes, it is a mature and simple technique to change the content of exosomes. Therefore, knockdown or overexpress the expression of certain microRNAs in exosomes is a significant step to confirm its function. As the contents of circulating exosomes could not be interfered from their source, we chose electroporation to directly transfect microRNAs into circulating exosomes with reference to relevant studies.^13^ We verified the success of this transfection method by fluorescent labelling microRNAs (Figure S1). In order to clarify the role of miR‐193a‐5p, we directly transfected miR‐193a‐5p mimics into AMI‐Exo. And animal experiment showed that AMI‐Exo transfected with miR‐193a‐5p mimics had a better protective effect (Figure 4E). To explore the downstream of miR‐193a‐5p, bioinformatics analyses including GO and KEGG by Metascape were applied.^36^ Through TargetScan and miRDB databases of microRNAs, we obtained 144 and 249 predicted target genes, respectively. The coincident predicted target genes in the above results, a total of 78 genes, were incorporated into GO and KEGG analyses to identify the possible downstream target of miR‐193a‐5p (Figure S2). The results showed that plasma membrane adhesion molecules managing homophilic cell adhesion were the most related targets (Figure 5A‐B). Among these predicted target genes, activin A receptor type I (ACVR1) and its downstream pathways had been shown to be involved in a variety of physiological and pathological processes.^18^ We found that the expression of ACVR1 was inhibited by miR‐193a‐5p transfection, and the dual‐luciferase report confirmed that ACVR1 was the target gene of miR‐193a‐5p (Figure 5C‐E). ACVR1 was a member of the BMP/TGFβ receptor family. It formed the transmembrane receptor complexes with type II receptors including BMPR2, ACVR2A and ACVR2B. The downstream of ACVR1 included SMAD1/5/8 and non‐SMAD signal transduction pathways like Rho, PI3K and P38.^37^, ^38^, ^39^, ^40^ Many studies had shown that ACVR1 is an important tumour suppressor participating in the biological processes of cancer cells like proliferation, migration, invasion, metastasis and apoptosis. Overexpression of ACVR1 in glioblastoma nude mice models lead to impeded tumour growth and longer survival. Activation of the ACVR1‐Smad1 signal pathway inhibited prostate cancer motility^42^ ACVR1 was also involved in the development of cardiovascular system. It had been reported that ACVR1 is required for the proper induction of endothelial to mesenchymal trans‐differentiation in atrioventricular cushion formation.^41^ ACVR1‐related signal pathway regulated vessel formation. Benn et al. reported that knockdown of ACVR1/ALK2 promoted sprouting of HUVECs, showing the potential effect on angiogenesis. ALK2 significantly inhibited the percentage of nuclei in tip cell position, which is important for sprout formation^43^ To verify that inhibiting the expression of ACVR1 can protect HUVECs from oxidative stress damage, an inhibitor of ACVR1/ALK2, ALK2‐IN‐2, was used. The results showed that ALK2‐IN‐2 and miR‐193a‐5p had consistent effects on the expression of ACVR1 and protection for endothelial cells (Figure 6A‐C). Overexpression of ACVR1 could reverse the vascular protective effects of miR‐193a‐5p, which indicated that miR‐193a‐5p acted as a protector by inhibiting ACVR1 (Figure 6D‐F).

**FIGURE 7:**
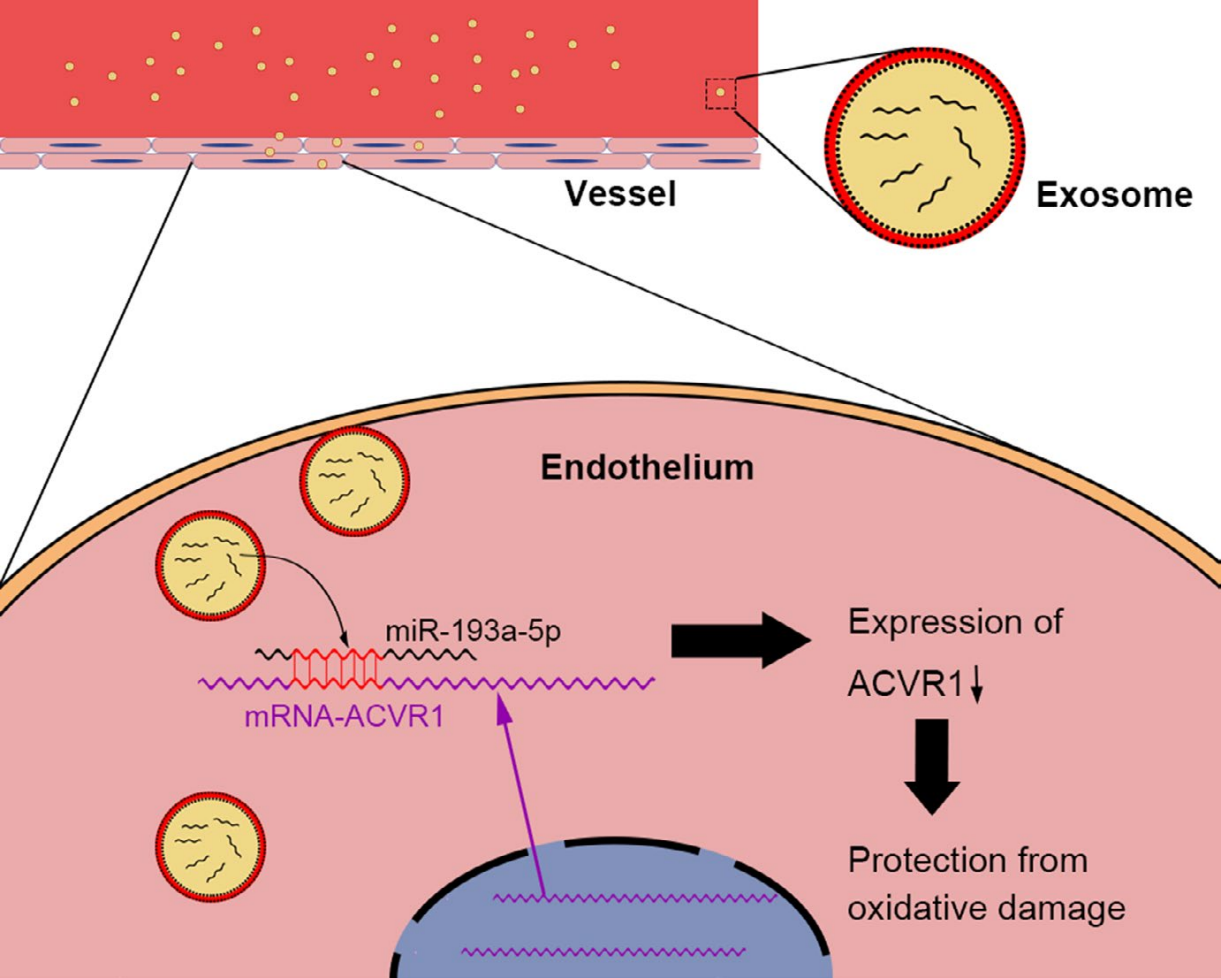
Circulating exosomes possible mechanism of protective effects on oxidative stress damage. Circulating exosomes could shuttle between cells through dynamin and delivery miR‐193a‐5p to protect endothelial cells from oxidative stress damage via ACVR1 signal pathway

In our study, we demonstrated that circulating exosomes could deliver miR‐193a‐5p to protect endothelial cells from oxidative stress damage via targeting ACVR1(Figure 7).

## 5 LIMITATIONS

However, our study still has some limitations. (a) The number of clinical samples (patients or healthy controls) included in this study was relatively small. Also in vitro studies, experiments were repeated 3 times for statistical significance, and the relatively small number of repetitions was one of the limitation of this study; (b) the source of circulating exosomes and the organizational origin of miR‐193a‐5p need to be further explored; (c) there was a lack of study on the upstream influence factors of miR‐193a‐5p, which is the focus of our future research; (d) compared with healthy people, hospitalized patients with another illness could eliminate the influence of the other factors including hospitalization, stress status, medication on exosomal status. Therefore, hospitalized patients with less than 50% coronary artery stenosis would be a better control group; (e) Compared with HUVECs, human coronary artery endothelial cells (HCAECs) were more specific and suitable to our study. HUVECs were widely used in cardiovascular research and had accumulated a wealth of evidence. Because of the limited conditions, we used HUVECs for in vitro experiments.

## 6 CONCLUSION

In conclusion, we demonstrated that circulating exosomes could shuttle between cells through dynamin and deliver miR‐193a‐5p to protect endothelial cells from oxidative stress damage via targeting ACVR1.

## CONFLICT OF INTEREST

The authors confirmed that there was no conflict of interest.

## AUTHORS’ CONTRIBUTION

CC: Conceptualization (Equal); Data curation (Equal); Formal analysis (Equal); Investigation (Equal); Methodology (Equal); Writing—original draft (Equal). BW: Data curation (Equal); Formal analysis (Equal); Investigation (Equal); Methodology (Equal); Writing—original draft (Equal). JT: Conceptualization (Equal); Funding acquisition (Equal); Project administration (Equal); Supervision (Equal); Validation (Equal); Visualization (Equal); Writing—review and editing (Equal). JZ: Investigation (Equal); Methodology (Equal); Visualization (Equal). JG: Investigation (Equal); Methodology (Equal). GQ: Formal analysis (Equal); Investigation (Equal). XY: Investigation (Equal); Visualization (Equal). ZZ: Formal analysis (Equal). GL: Formal analysis (Equal). HZ: Investigation (Equal); Methodology (Equal). YW: Formal analysis (Equal). JZ: Conceptualization (Equal); Funding acquisition (Equal); Project administration (Equal); Writing—review and editing (Equal).

## Supporting information

Fig S1

Fig S2

## Data Availability

The data sets generated and/or analysed during the current study are available from the corresponding author upon reasonable request.
