# Supplementary figures and images for: Circulating exosomes repair endothelial cell damage by delivering miR‐193a‐5p
Source: J Cell Mol Med. 2020 Dec 22;25(4):2176–89. doi: 10.1111/jcmm.16202 (PMC7882992; doi:10.1111/jcmm.16202)

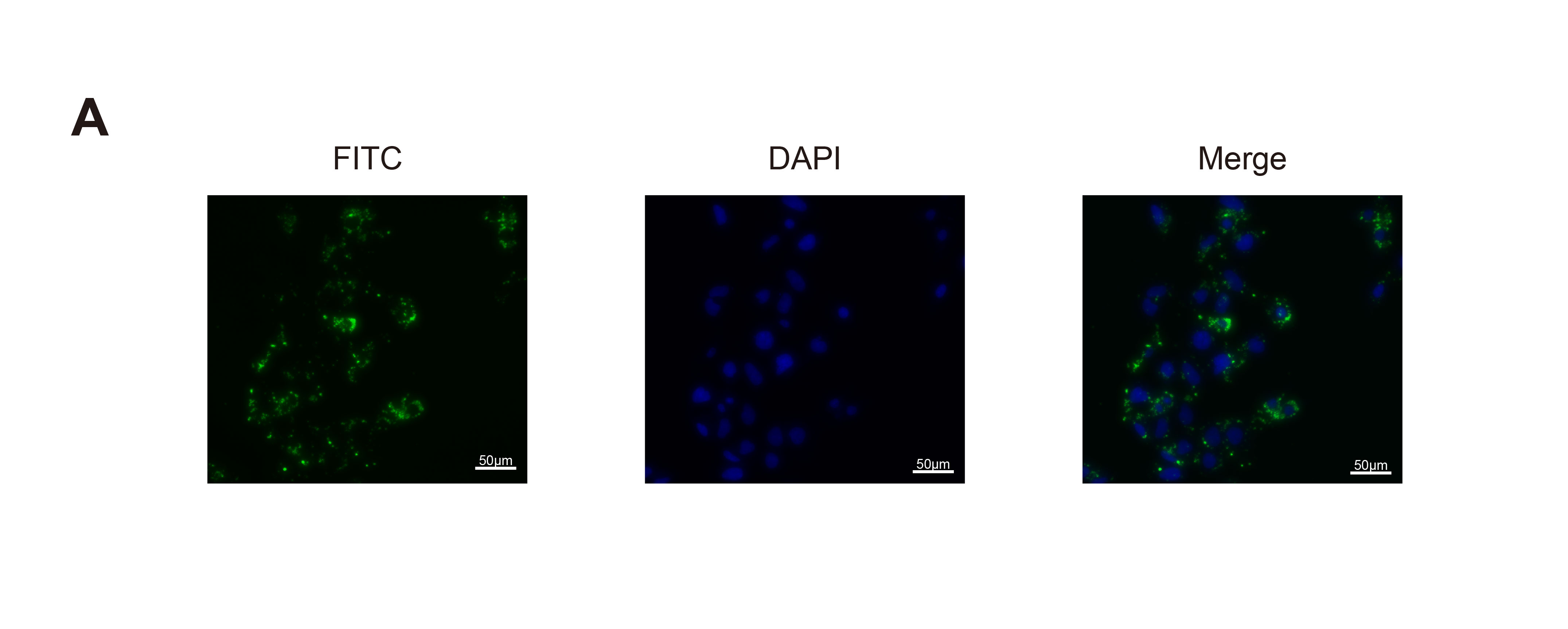

Supplement: Supplementary file 1 — Fig S1 [file JCMM-25-2176-s001.jpg]

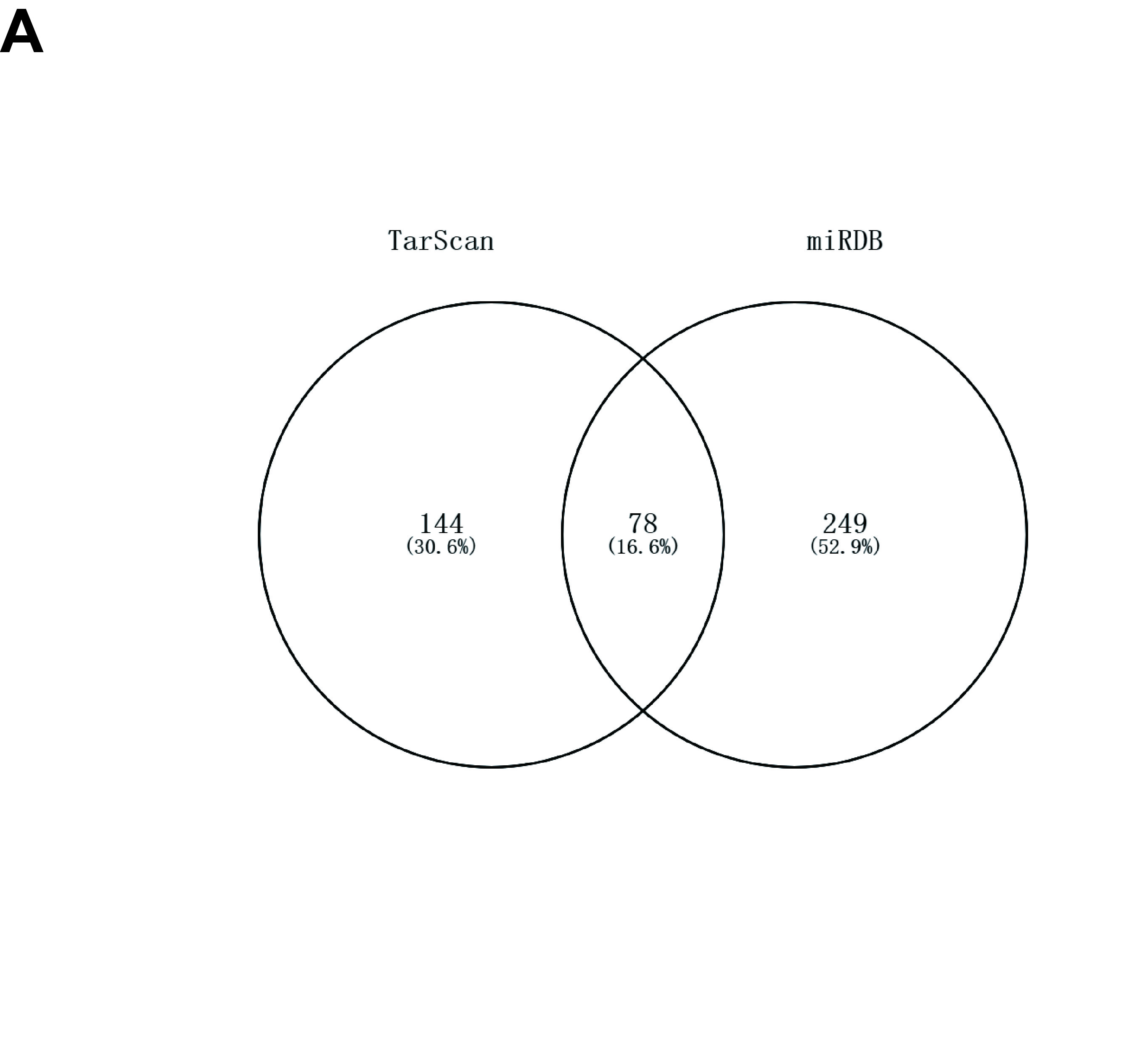

Supplement: Supplementary file 2 — Fig S2 [file JCMM-25-2176-s002.jpg]
